# Effects of Probiotic Fermented Fruit Juice-Based Biotransformation by Lactic Acid Bacteria and *Saccharomyces boulardii* CNCM I-745 on Anti-Salmonella and Antioxidative Properties

**DOI:** 10.4014/jmb.2206.06012

**Published:** 2022-09-16

**Authors:** Wanida Laosee, Duangporn Kantachote, Worrapanit Chansuwan, Nualpun Sirinupong

**Affiliations:** 1 Functional Food and Nutrition Program, Faculty of Agro-Industry, Prince of Songkla University, Hat Yai, Songkhla 90110, Thailand; 2 Department of Microbiology, Faculty of Science, Prince of Songkla University, Hat Yai, Songkhla 90110, Thailand; 3 Center of Excellence in Functional Foods and Gastronomy, Prince of Songkla University, Hat Yai, Songkhla 90110, Thailand

**Keywords:** Biotransformation, fermentation, probiotic, lactic acid bacteria, yeast, fruit juice

## Abstract

Fermentation is an effective process for providing various beneficial effects in functional beverages. Lactic acid bacteria and yeast fermentation-based biotransformation contribute to enhancement of nutritional value and digestibility, including lactose intolerance reduction and control of infections. In this study, the probiotic fermented fruit juice (PFJ) was produced by *Lactobacillus plantarum* TISTR 1465, *Lactobacillus salivarius* TISTR 1112, and *Saccharomyces boulardii* CNCM I-745 while mixed fruit juice (MFJ) was used as the basic medium for microorganism growth. The potential function, the anti-salmonella activity of PFJ, was found to be effective at 250 mg/ml of MIC and 500 mg/ml of MBC. Biofilm inhibition was performed using the PFJ samples and showed at least 70% reduction in cell attachment at the MIC concentration of *Salmonella* Typhi DMST 22842. The antioxidant activities of PFJ were determined and the results revealed that FSB.25 exhibited 78.40 ± 0.51 mM TE/ml by FRAP assay, while FPSB.25 exhibited 3.44 ± 0.10 mM TE/ml by DPPH assay. The volatile compounds of PFJ were characterized by GC-MS, which identified alcohol, aldehyde, acid, ester, ketone, phenol, and terpene. The most abundant organic acid and alcohol detected in PFJ were acetic acid and 2-phenylethanol, and the most represented terpene was β-damascenone. The sensory attributes showed scores higher than 7 on a 9-point hedonic scale for the FPB.25, illustrating that it was well accepted by panelists. Taken together, our results showed that PFJ could meet current consumer demand regarding natural and functional, fruit-based fermented beverages.

## Introduction

Recently, consumer awareness of healthy foods and eating habits has generated a massive market demand for functional foods with health benefits. Fermented foods, particularly non-dairy beverages, are gaining popularity and acceptance due to their functional benefits [1].

Biotransformation based on fermentation is controlled by two main factors, microorganisms and substrates [2]. Lactic acid bacteria (LAB) and yeast are the most used probiotics in fermented foods. Some species of yeast, including *Saccharomyces cerevisiae* var. *boulardii* (*Saccharomyces boulardii*), and LAB, such as *L. plantarum* and *L. salivarius*, showed positive probiotic results in the inhibition of pathogenic bacteria growth and antibiotic resistance [3]. To improve the nutritional value and organoleptic characteristics of fruit fermented juice, mixing of yeast and LAB as a multi-strain starter may provide better beneficial effects than mono-strain culture [4]. During the fermentation process, both yeast and LAB are the two main microbial groups involved in the ester and terpene production. Yeast is responsible for alcoholic fermentation and determines the production of major alcohol and esters [5]. The components of alcoholic beverages can be split into major and minor groups. The major group consists of ethanol and water, and the minor group comprises fusel alcohols, carbonyl compounds, esters, organic acids, aldehydes, lactones, and sulfur compounds [6]. Through biotransformation, LAB can produce the volatile flavors of fermented foods, which are composed of organic acids, alcohols, and ketone and aldehyde compounds [7]. When LAB and yeast were cocultured, they enhanced the growth of both groups as well as the flavor, thereby improving the organoleptic properties of fermented products [8].

The survival rate of probiotics during exposure to strong adverse conditions in the gastrointestinal tract is fundamental and may be affected by the components in the fermented beverages [9]. Moreover, a high survival rate of probiotics during production, handling, and storage of beverages is critical to obtain the desired health benefits for the consumer [10]. Fruit juices have been studied and reported to be an appropriate medium for probiotic fermented beverages and the merging of nutritional value with the added probiotics [11]. Therefore, probiotic fruit beverages are being produced in large volume due to an increased demand for functional foods.

Although several studies have reported on the influence of probiotics on the quality and functionality of fermented fruit juice, there is scant data supporting the improvement of antioxidant and volatile compounds in fermented fruit beverages. In this study, two main crops, *Benincasa hispida* Cogn and *Ananas comosus* L. Merr., were chosen as beverage components. The effect of two selected commercial LAB strains and yeast fermentation in the biotransformation was determined as well as the growth culture improvement, sugar consumption, and organic acid production during fermentation. Moreover, the antibacterial properties and the changes in antioxidant capacities were analyzed.

## Materials and Methods

### Microorganisms and Culture Conditions

The LAB strains *L. plantarum* TISTR 1465 (P) and *Lactobacillus salivarius* TISTR 1112 (S), as well as the commercial yeast strain *S. boulardii* CNCM I-745 (B), were obtained from the Thailand Institute of Scientific and Technological Research (TISTR). The LAB and yeast were maintained at -20°C in de Man, Rogosa and Sharpe (MRS) broth and yeast malt (YM) broth supplemented with 30% glycerol (v/v), respectively. For stimulation, 1 ml of the cultures was added into 50 ml of MRS broth (LAB) and YM broth (yeast) and incubated at 37°C for 24 h. Then, the cell suspension was centrifuged at 6,000 ×*g*, 4°C for 10 min, after which the cell pellet was washed twice using sterile water. The optical density (OD_660_) was measured, and suitable dilutions were made using sterile water to gain an OD_660_ of 1.5 and *S. boulardii* (B) cells of approximately 7 log CFU/ml, and an OD_660_ of 2.5 to obtain LAB of approximately 8 log CFU/ml. The activated cell suspensions were used for inoculation in the fermentation medium.

### Preparation of MFJ and Fermentation

Winter melon (*Benincasa hispida* Cogn.) and pineapple (*Ananas comosus* L. Merr.) were obtained from a local market in southern Thailand. Longan (*Dimocarpus longan* Lour.) honey was obtained from beekeepers. The ratio of the constituents for the mixed fruit juice (MFJ) was an appropriate proportion (v/v) of pineapple juice, winter melon juice and longan honey as described in a previous study [12]. The pH was adjusted to 5.4 ± 0.5. MFJ pasteurized at 70°C for 10 min was cooled down to 37°C. Single-culture fermentations by LAB strain *L. plantarum* (P) or *L. salivarius* (S) were carried out with 1% (v/v) inoculation, or approximately 6 log CFU/ml. Then, sequential mixed fermentation was performed by inoculation with 1% (v/v) of activated LAB cells, followed 48 h later with 0.25% (v/v), approximately 4 log CFU/ml, of activated *S. boulardii* (B) cells. All of the above 900-ml formulas were incubated at 37°C for 72 h, following inoculation with pre-cultures of LAB or yeast. Uninoculated MFJ was taken as control and other fermented frut juices inoculated with different proportion of microorganisms were coded in the whole manuscript as follows: FP, fermented with 1% *L. plantarum* (P); FS, fermented with 1%*L. salivarius* (S); FPB.25, fermented with 1% *L. plantarum* (P) and 0.25% *S. boulardii* (B); FSB.25, fermented with 1% *L. salivarius* (S) and 0.25% *S. boulardii* (B); FPSB.25, FPB.25 and FSB.25 mixed at a ratio of 1:1.

### Determination of Viable Cell Counts

Viable cell count of probiotics in fermented fruit juice (PFJ) was performed by the spread plate method using serial dilution with 0.85% NaCl to 10^5^-10^7^ dilutions. An aliquot of 0.1 ml of each dilution was spread in triplicate on media of MRS agar and YM agar (supplemented with 100 mg penicillin G). The plates were then placed in the incubator at 37°C for 24-48 h, and the total numbers of LAB and *S. boulardii* were determined by colony counting. Plates containing 25-250 colonies were counted and marked as log CFU/ml [13].

### Determination of Physicochemical Properties

The titratable acidity was measured by 0.1 M NaOH titration and expressed as the percentage of lactic acid [14]. The alcohol content and pH were determined by ebulliometer (Dujardin-Salleron, France) and pH meter (Horiba F22, USA), respectively. Reducing sugar and total sugar contents were analyzed based on glucose equivalents of the 3, 5-dinitrosalicylic acid [15] and phenol sulfuric acid methods [16], respectively. Absorbance was determined by UV-Vis spectrophotometer (Agilent BioTek, USA).

### Determination of Total Phenolic Content

Total phenolic content of PFJ sample was determined by the Folin-Ciocalteu method according to [17] with modifications. Briefly, 60 μl of fresh 10% Folin reagent was added to 20 μl of diluted sample, and after 1 min, 60 μl of 7.5% Na_2_CO_3_ was added into the mixture followed by 60 ml of distilled water and incubation at RT for 30 min. The OD_760_ was measured by spectrophotometer (Agilent BioTek, USA). The results were presented as milligrams of gallic acid equivalent per milliliter (mg GAE/ml) of sample.

### Determination of Antioxidative Activity

Antioxidative activity of PFJ sample was determined by DPPH radical scavenging activity and FRAP activity. The DPPH assay was performed as described by [18]. Briefly, 1.8 ml of 200 μM DPPH solution was combined with 200 μl of sample. The combination was incubated in the dark at RT for 30 min. The OD_517_ was measured for the ability of DPPH of the sample. The obtained values were calculated according to the Trolox equivalent antioxidant capacity (TEAC) standard (ml/mM).

The FRAP assay was performed by [19] with some modifications. Briefly, the reaction was mixed with the following solution: 10 ml of 40 mM 2, 4, 6-tris (2-pyridyl)-1, 3, 5-triazine solution, prepared with 40 mM HCl, and 10 ml of 20 mM FeCl_3_.6H_2_O, 100 ml of 0.3 M acetate buffer pH 3.6. Then, 200 μl of the PFJ sample was added into 1.8 ml of the reaction mixture and incubated at RT for 30 min. The OD_593_ was measured and the activity by the FRAP assay was expressed using the Trolox standard (ml/mM).

### Determination of Anti-*Salmonella* Potential and Inhibition of Biofilm Formation

The anti-salmonella potential, evaluated by the minimum inhibitory concentration (MIC) of the PFJ, was performed by serial dilution method according to the guidelines of the Clinical and Laboratory Standard Institute (CLSI) procedures [20]. Two-fold serially diluted PFJ with brain heart infusion (BHI) was prepared using sterilized 96-well plates. The diluted PFJ was mixed with 100 μl of *Salmonella* Typhi DMST 22842 cultures (approximately 10^8^ CFU/ml) ranging from 62.5-500 mg/ml. Gentamicin (0.039-1.25 mg/ml) was used as positive control. The plates were incubated at 37°C for 24 h. The lowest concentration of sample inhibiting bacterial growth, which is the definition of MIC, was observed. For determination of minimum bactericidal concentration (MBC), the 10 ul aliquots from the previous MIC result were added into BHI agar, and then incubated at 37°C for 24 h. The lowest concentration of PFJ inhibiting pathogen growth by 99.9% (with no growth observed) was the MBC value.

The inhibition of biofilm formation was determined using the method of [21] with some modifications. First, 100 μl of PFJ sample from the MIC was added into 96-well plates. Then, 100 μl of gentamicin and distilled water were used as positive control and negative control, respectively. After that, 100 μl of *Salmonella* Typhi DMST 22842 culture (approximately 10^8^ CFU/ml) was added to a 200 μl final volume. The plates were placed at 37°C for 24 h. Following that, the supernatant was discarded and the formed biofilm was washed using distilled water. Then, the plates were fixed at 60˚C for 1 h. Finally, the biofilm was stained using 0.1% crystal violet/water. After staining, the plates were washed twice using sterile water. The biofilm was quantified by applying 30% acetic acid solution (200 μl) to observe the biofilm formation. The OD_595_ was measured. The inhibition rate of the biofilm was calculated as follows: I% = [(A_C_ – A_T_)/A_C_ × 100], where AC is the OD_595_ of control growth and AT is the OD_595_ of treated growth.

### Determination of Volatile Compounds

Volatile compounds of the PFJ product were characterized using an HS-SPME/GC-MS system according to the previously reported method [22] with some modification. Briefly, 1.8 ml of PFJ was put into a glass vial and the headspace was incubated at 40°C for 30 min, with an equilibration time of 15 min. Before the analyses, the fiber was inserted in the injector at 230°C for 2 min and the desorption of volatiles was accomplished by exposing the fiber at 230°C for 2 min. The separation was performed in a VF-WAXms capillary column at temperatures starting at 50°C for 3 min, and then increased by 5°C/min to 200°C and maintained for 12 min. The line temperature was 250°C. The signal acquisition was full scan from 41 m/z to 500 m/z. The volatile compounds produced in the PFJ were identified based on a mass spectral library match of 90% (NIST 14).

### Sensory Evaluation

The PFJ products were evaluated for organoleptic properties using 50 untrained panelists. A short explanation on the attributes and the 9-point hedonic scale, ranging from dislike extremely (1) to like extremely (9), was provided [23]. Consumers evaluated the product for appearance, color, sour odor, fruity odor, sour taste, fruity flavor, sweetness, and overall acceptability. The PFJ was served in 20 ml plastic cups and water was used in between to eliminate the sample flavor interactions. The data of all characteristics were calculated and plotted.

### Statistical Analysis

Measurements were carried out in triplicate and the values were calculated as means ± standard deviation. One-way analysis of variance (ANOVA) and Tukey’s test were performed using SPSS 21 to evaluate differences among groups. A value of *p* < 0.05 was considered statistical significance.

## Results and Discussion

### Viable Cell Counts

**Fig. 1:**
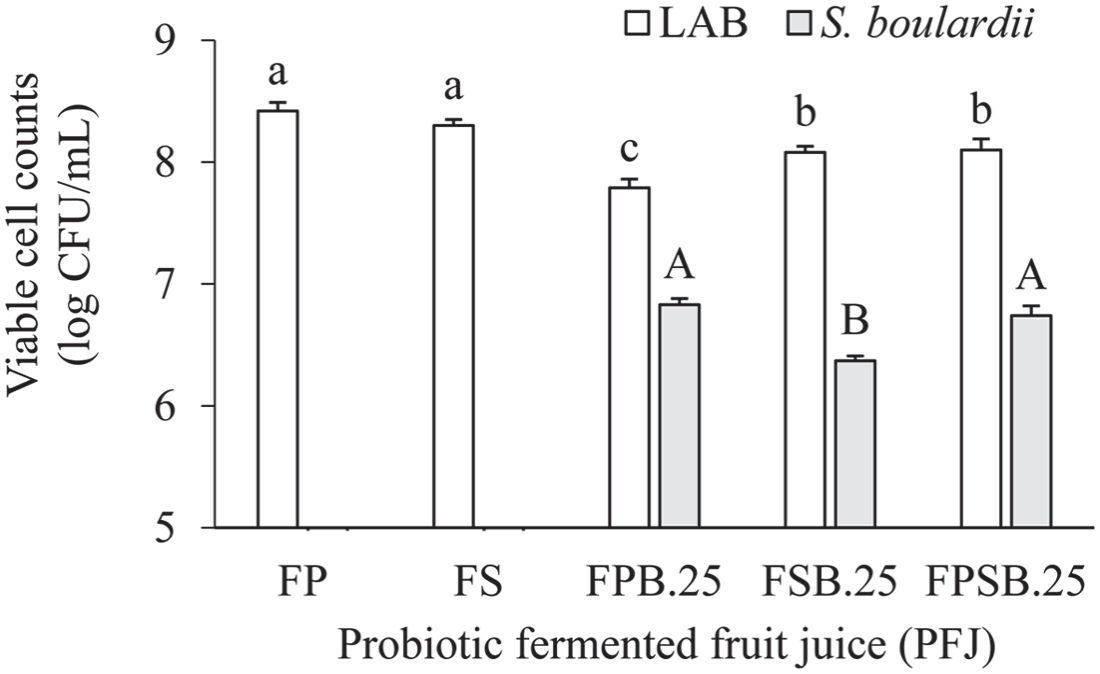
Viable cell counts of lactic acid bacteria (LAB) and *S. boulardii* of probiotic fermented fruit juice (PFJ). FP, fermented with 1% *L. plantarum* (P); FS, fermented with 1% *L. salivarius* (S); FPB.25, fermented with 1% *L. plantarum* (P) and 0.25% *S. boulardii* (B); FSB.25, fermented with 1% *L. salivarius* (S) and 0.25% *S. boulardii* (B); FPSB.25, mixed FPB.25 and FSB.25 at a ratio of 1:1. Results are expressed as means ± SD (*n* = 30), Different letters in the same fermented microorganism indicate statistical significance difference (*p* < 0.05).

The cell viability of LAB and yeast *S. boulardii* in PFJ after fermentation was shown in Fig. 1. A similar increase of LAB was observed significantly (*p* > 0.05) during single-culture fermentation of FP and FS, at around 8.35 log CFU/ml. Meanwhile, cell viability of LAB was significantly decreased (*p* < 0.05) in the combined probiotic with *S. boulardii* in FPB.25 and FSB.25 to 7.79 ± 0.07 and 8.08 ± 0.05 log CFU/ml, respectively. However, the results showed that cell viability of LAB at the end of the fermentation process above the threshold suggested that a fermented product could provide a therapeutic effect of 6 log CFU/ml based on a 100 ml sample of a product containing live probiotic [24]. Regarding yeast, the FPB.25 and FSB.25 exhibited a significant (*p* < 0.05) increase in cell viability of the yeast *S. boulardii* population during mixed culture fermentation of 6.83 ± 0.05 and 6.37 ± 0.04 log CFU/ml, respectively. Additionally, the FPSB.25 showed LAB and yeast populations of 8.10 ± 0.09 and 6.74 ± 0.08 log CFU/ml, respectively (Fig. 1). Microorganisms in mixed culture fermentation may compete for nutrients or may generate metabolites that stimulate or inhibit each other’s growth. Previous studies reported that the growth of yeast in fermented foods is favored by acidification of the environment created by LAB [25], and the yeast may provide growth factors such as vitamins and soluble nitrogen compounds to stimulate the growth of LAB [26].

### Physicochemical Properties

The changes in sugar content, titratable acidity, pH and alcohol content after fermentation of PFJ are presented in Table 1. The pH value was significantly decreased (*p* < 0.05) from 5.55 to 3.75 after fermentation. The reducing of pH showed indicates production of organic acids during LAB fermentation [27]. It was also found that the titratable acidity significantly increased (*p* < 0.05) in PFJ comparable to MFJ. Typically, the pH values showed an opposite trend to that observed for titratable acidity. As seen in Table 1, a decrease of pH value was observed when the acidity increased. Sugar content of PFJ was evaluated (total sugar and reducing sugar). The amount of total sugar and reducing sugar in MFJ was 1825.81 ± 12.33 and 0.80 ± 0.03 g/l, respectively, and after fermentation, they were both significantly decreased (*p* < 0.05) (Table 1). The decrease of sugar contents may be the result of the biotransformation to organic acid and the utilization for metabolism and growth of LAB strains [28]. During fermentation, *L. plantarum* acts as facultative, hetero-fermentative LAB capable of utilizing glucose and sucrose via the pentose phosphate pathway and producing different metabolized end products such as lactic acid, acetic acid, and carbon dioxide, similar to other hetero-fermentative bacteria, or only produces lactic acid depending on the type of carbohydrate metabolism available for fermentation [29]. Meanwhile, *L. salivarius* is an obligatory homo-fermentative anaerobe that mostly produces a single, metabolized end product (lactic acid) from carbohydrate metabolism by the Embden-Meyerhof-Parnas (EMP) pathway [30]. Alcohol content was approximately 0.29 to 0.36% in the PFJ of mixed culture fermentation with *S. boulardii*. The yeast *S. boulardii* employs respiro-fermentative metabolism in which a combination of glycolysis and the pentose phosphate pathway was utilized for hexose metabolism [31].

### Total Phenolic Content

**Fig. 2:**
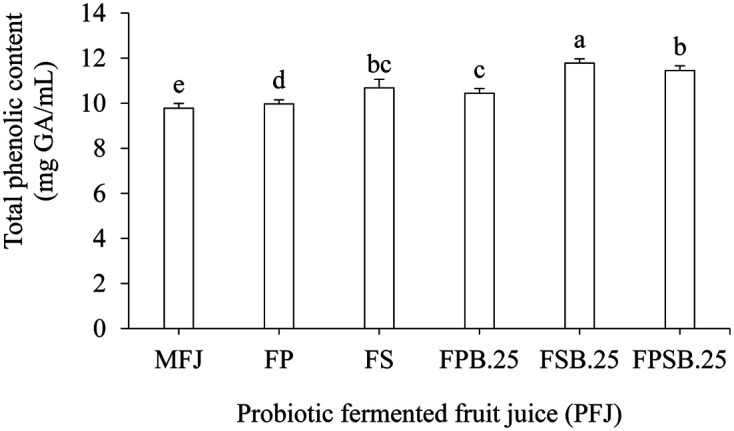
Total phenolic content of probiotic fermented fruit juice (PFJ). MFJ, Mixed fruit juice control; FP, fermented with 1% *L. plantarum* (P); FS, fermented with 1% *L. salivarius* (S); FPB.25, fermented with 1% *L. plantarum* (P) and 0.25% *S. boulardii* (B); FSB.25, fermented with 1% *L. salivarius* (S) and 0.25% *S. boulardii* (B); FPSB.25, mixed FPB.25 and FSB.25 at a ratio of 1:1. Results expressed as means ± SD (*n* = 30). Different letters indicate significant difference (*p* < 0.05).

The total phenolic content of FSB.25 and FPSB.25 was increased significantly (*p* < 0.05) to 11.78 ± 0.19 and 11.45 ± 0.21 mg GA/ml, respectively, compared to MFJ (Fig. 2). The increase in the free form of phenolic content present in the fermented fruit juice might be metabolized and degraded small molecules, resulting in the phenolic content increase (Fig. 2). In addition, the LAB contains some enzymes that might aid in degrading phenolic compounds, which may also be a potential reason for the enhanced total phenolic content in the fermented fruit juice (Fig. 2) [32].

### Antioxidative Activity

**Fig. 3:**
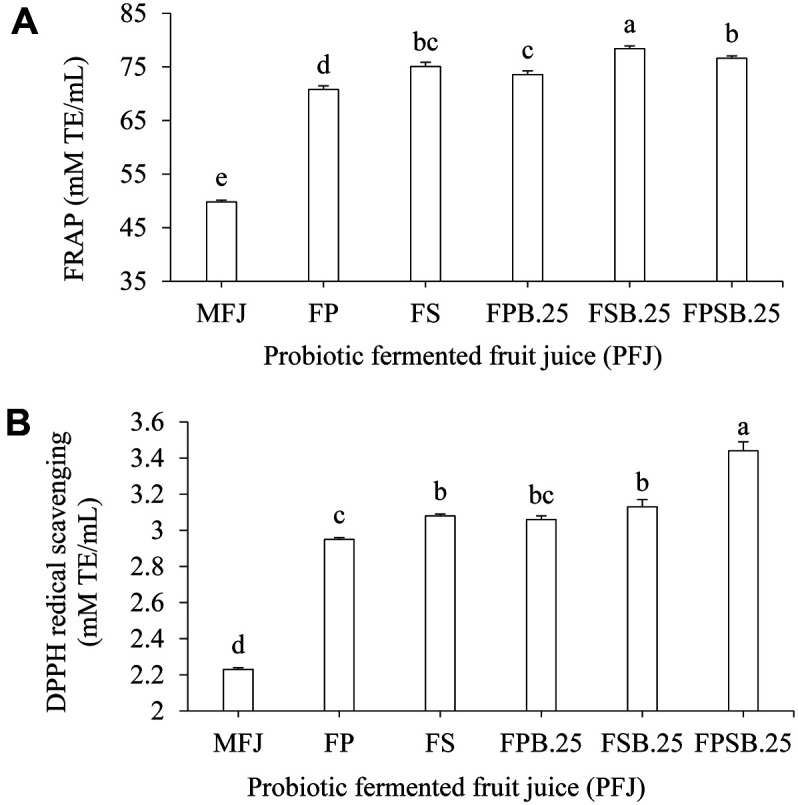
Antioxidant activity of probiotic fermented fruit juice (PFJ). FRAP activity (**A**) and DPPH activity (B) of MFJ, Mixed fruit juice control; FP, fermented with 1% *L. plantarum* (P); FS, fermented with 1% *L. salivarius* (S); FPB.25, fermented with 1% *L. plantarum* (P) and 0.25% *S. boulardii* (B); FSB.25, fermented with 1% *L. salivarius* (S) and 0.25% *S. boulardii* (B); FPSB.25, mixed FPB.25 and FSB.25 at a ratio of 1:1. Results expressed as means ± SD (*n* = 30). Different letters indicate significant difference (*p* < 0.05).

The antioxidative activity of PFJ determined by FRAP and DPPH assay was significantly (*p* < 0.05) higher than that of MFJ. In case of FSB.25, the FRAP assay showed a value of 78.65 ± 0.51 mM TE/ml (Fig. 3A). The increased antioxidant activity obtained in the FRAP assay may be related to the phenolic content, which is associated with the reduction of 2,4,6-tris (2-pyridyl)-S-triazine TPTZ-Fe^3+^ complex to TPTZ-Fe^2+^ [33]. Furthermore, the DPPH assay result showed a value increased to 3.44 ± 0.10 mM TE/mL for FPSB.25 compared with MFJ (Fig. 3B). The increased DPPH radical scavenging activity suggests that LAB fermentation might enhance the availability of phenolic compounds with proton-donating properties [34]. In addition, yeast has been shown to generate great numbers of antioxidative substances [35]. For example, the extracellular fraction extracted from *S. boulardii* is dense in polyphenolic metabolites like vitamin B_6_ and 2-phenylethanol, which revealed that significant antioxidative activity by the DPPH assay [36]. The increase of antioxidative activity demonstrates the positive health benefits of probiotic fermented fruit juice compared to a non-fermented product.

### Anti-Salmonella Potential and Inhibition of Biofilm Formation

**Fig. 4:**
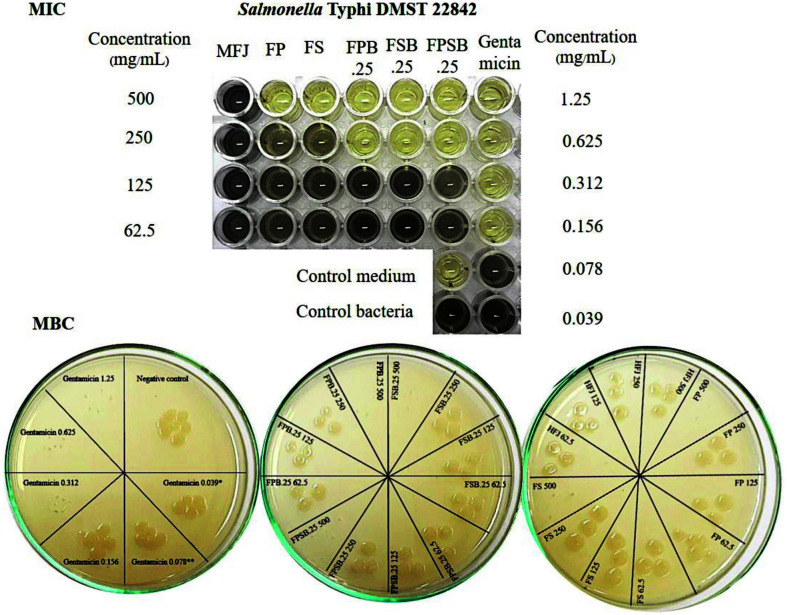
The anti-salmonella potential of MFJ and PFJ against *Salmonella* Typhi DMST 22842 based on MIC and MBC. MFJ, Mixed fruit juice control; FP, fermented with 1% *L. plantarum* (P); FS, fermented with 1% *L. salivarius* (S); FPB.25, fermented with 1% *L. plantarum* (P) and 0.25% *S. boulardii* (B); FSB.25, fermented with 1% *L. salivarius* (S) and 0.25% *S. boulardii* (B); FPSB.25, mixed FPB.25 and FSB.25 at a ratio of 1:1.

The anti-salmonella potential of MFJ and PFJ against *Salmonella* Typhi DMST 22842 assessed on the basis of their MIC and MBC were shown in Fig. 4. The antibacterial activities of PFJ fermented by yeast *S. boulardii* were found to be effective and showed MIC and MBC values of 250 and 500 mg/ml, respectively, while the MFJ was not able to inhibit *Salmonella* Typhi DMST 22842. The positive control was gentamicin, which exhibited MIC and MBC values of 0.039 and 0.078 mg/ml, respectively (Table 2). This growth inhibition could due to the antimicrobial compounds produced by LAB and yeast in the fermentation process. During fermentation, the anti-bacterial properties of organic and undissociated acid produced by probiotic microorganisms may enter into the cell and release protons in the cytoplasm resulting in reduction of pH in cytoplasm [37]. Additionally, undissociated acid results in substrate transport system destruction by destroying the electrochemical proton gradient or changing the permeability of the cell membrane [38]. Therefore, the anti-salmonella potential was improved.

**Fig. 5:**
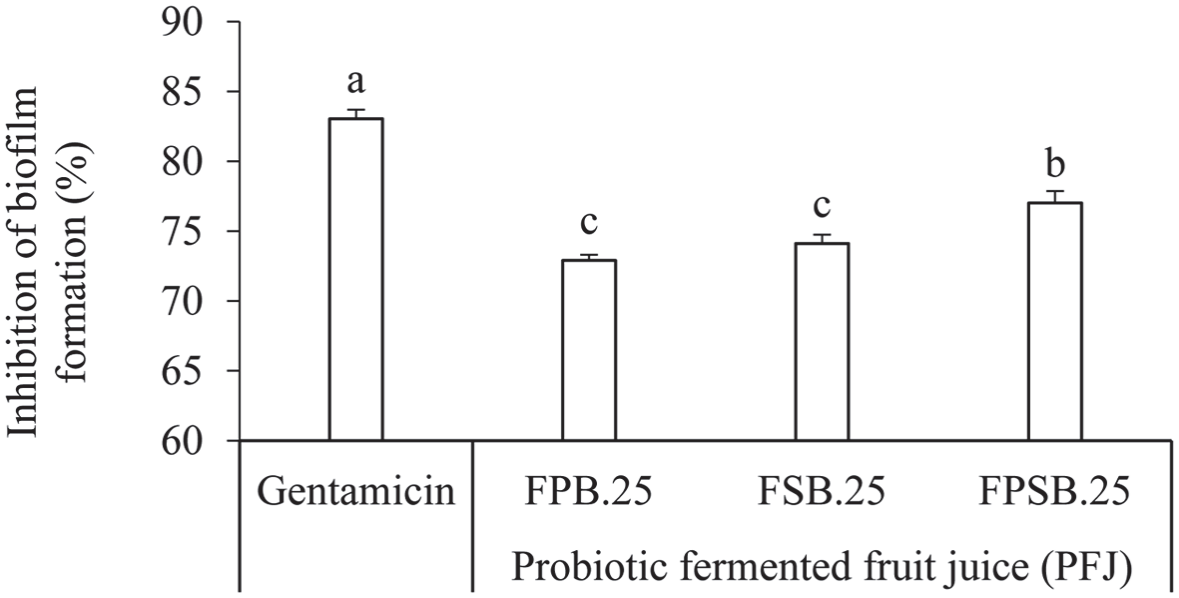
Inhibition of the biofilm formation for the MIC of probiotic fermented fruit juice (PFJ). FPB.25, fermented with 1% *L. plantarum* (P) and 0.25% *S. boulardii* (B); FSB.25, fermented with 1% *L. salivarius* (S) and 0.25% *S. boulardii* (B); FPSB.25, mixed FPB.25 and FSB.25 at a ratio of 1:1. Results expressed as means ± SD (*n* = 30). Values with different superscripts in the same pattern column indicate significant difference (*p* < 0.05).

Analysis of biofilm formation inhibition was conducted for the PFJ samples, which showed at least 70%reduction in MIC concentration in cell attachment of both tested *Salmonella* Typhi DMST 22842 by using the crystal violet method. The results exhibited different effects on the growth and development of biofilm formation as presented in (Fig. 5). The percentages of biofilm formation on the *Salmonella* Typhi strains were significantly (*p* < 0.05) inhibited in PFJ. Biofilm formation allows the bacteria integrated into the biofilm to be protected from environmental fluctuations such as temperature, humidity, and pH. In the case of infection, antibacterial preparation applied to the host organism might increase the period of infection, providing concentrated nutrients and waste-management mechanisms. Recently, studies have reported that bacteriocin from LAB strain may also inhibit biofilm formation. The effect of bacteriocin on sensitive bacterial strains is bactericidal while the bacteriocins of *L. plantarum*, such as plantaricin GZ1-27, have bactericidal effect [39].

### Volatile Compound Identification

Volatile compounds are one of the most important characteristics of beverage products for consumer palatability. A total of 14 volatile compounds identified in MFJ and PFJ were produced by LAB strains and *S. boulardii* and presented in Table 3. The compounds were characterized by class as follows: alcohol (3), aldehyde (1), acid (1), ester (5), ketone (1), phenol (1) and terpene (2). Alcohols are important aromatic compounds of yeast and LAB fermentation and contribute light aroma. The 2-phenylethanol, which positively affects beverage aroma and is associated with flowery, honey-like odor [40], could be produced from amino acid metabolism (Ehrlich pathway) and metabolism of sugar in yeast [41]. Moreover, 3-methyl-1-butanol is generated by reduction of the associated aldehydes obtained from BCAA metabolism, as in the case of leucine [42]. One important identified volatile compound in MFJ, 1-hexanol, was the most important alcohol and contributed to the sweetness sensation. The production of 1-hexanol occurred during the linoleic acid oxidation process [43]. The most abundant alcohol detected in PFJ was 3-methyl-1-butanol. In addition, the highest amount of 3-methyl-1-butanol was detected in the FPSB.25 sample. However, these alcohol compounds likely contribute to the flavor and odor-detection thresholds.

Acetaldehyde was found to be the major alcohol in the aldehyde group. Acetaldehyde is generated by LAB and yeast, the main intermediate in ethanol production. Acetaldehyde provides an attractive fruity aroma when present in low concentrations; however, it exhibits a pungent odor at high concentration [44]. Acid compounds, which are the main cause of sour and acid odors with high amounts of acetic acid, were detected in the PFJ of FPB.25. The production of acid was associated with carbohydrate metabolism or lipid degradation. Thus, the release of large quantities of acetic acid indicated that LAB strain *L. plantarum* could metabolize lactic acid [45].

Among the volatile esters identified were those mainly due to the medium-chain fatty acid (MCFAs), which are produced by the combination of MCFAs-CoA with ethanol [46]. The dominant esters of MFJ were ethyl 2-methylbutyrate and ethyl acetate, which have the odor of fruit and pineapple. Interestingly, the ethyl 2-methylbutyrate and ethyl acetate in PFJ were decreased during the fermentation process, probably because the volatilization or ester hydrolysis was higher during their formation [47]. The evolution trend of ethyl decanoate varied during the fermentation process, while that of ethyl hexanoate exhibited an increase at the early stage of the fermentation process. The main esters present, such as ethyl hexanoate and ethyl lactate, are characterized by a fruity and sweet odor. The major volatile ketone identified in PFJ was 3-hydroxy-2-butanone and could be biosynthesized from citrate, which has a buttery odor [48]. As for phenolic compounds, which are important volatile organic compounds in the MFJ and PFJ, 2, 4-di-tert-butylphenol was among those identified. Among the class of terpenes were linalool and β-damascenone. The β-damascenone production was attributed to glycosylases in LAB strains, which could cleave the bond between terpenes and sugars [49] and have a sweet and fruity odor.

### Sensory Evaluation

**Fig. 6:**
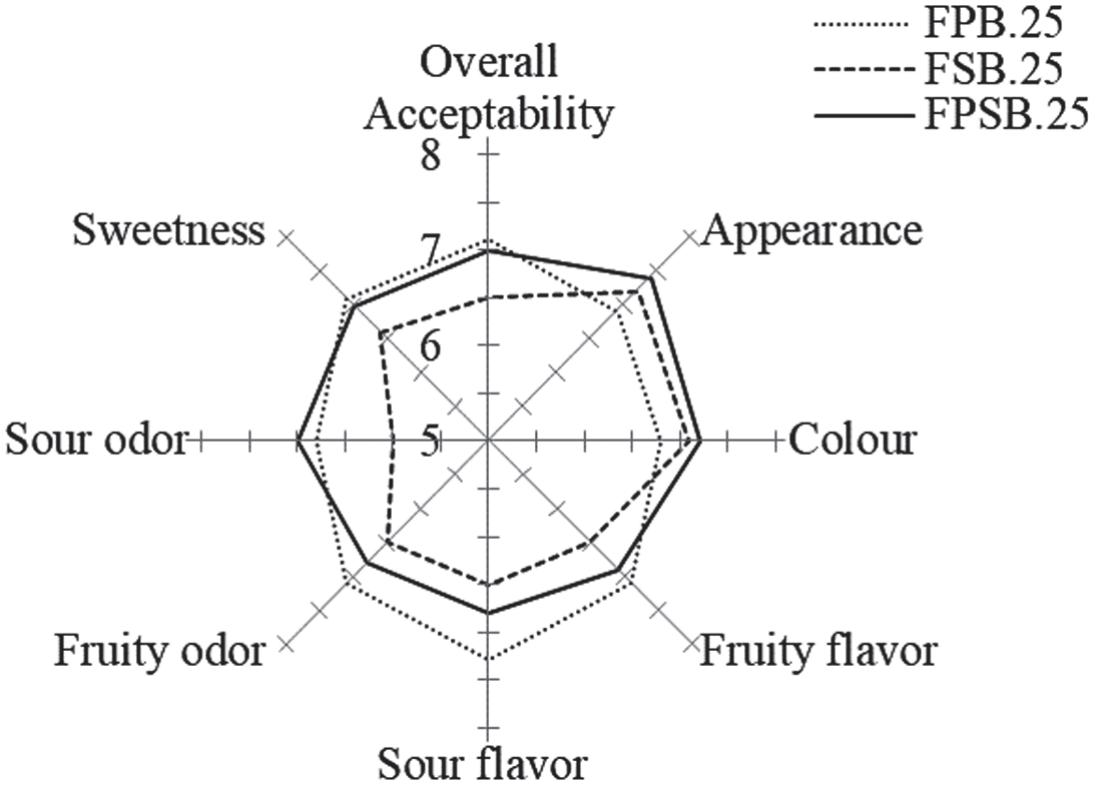
Sensory evaluation scores of probiotic fermented fruit juice (PFJ). FPB.25, fermented with 1% *L. plantarum* (P) and 0.25% *S. boulardii* (B); FSB.25, fermented with 1% *L. salivarius* (S) and 0.25% *S. boulardii* (B); FPSB.25, mixed FPB.25 and FSB.25 at a ratio of 1:1. Results are expressed as means ± SD (*n* = 50). Values with different superscripts in a column represent statistical data significance (*p* < 0.05) according to Tukey’s test. Acceptability scores were evaluated using a 9-point hedonic scale ranging from 1 (dislike extremely) to 9 (like extremely).

The sensory scores for the PFJ were provided by 50 untrained panelists and were evaluated according to seven attributes, including appearance, color, fruity flavor, sour flavor, fruit odor, sour odor, and sweetness, as shown in Fig. 6. The results revealed significant (*p* < 0.05) differences for sour odor and sour flavor between FPB.25 and FSB.25. Mixed fruit juice fermentation using *L. plantarum* strain showed the highest score for sour flavor at 7.3 ± 0.9 for the FPB.25 formula. Nevertheless, the overall acceptability of mixed culture fermentations by LAB and yeast *S. boulardii* did not have significantly (*p* < 0.05) different values, and showed 7.0 ± 1.1, 6.5 ± 1.5, and 6.7 ± 1.1 for FPB.25, FSB.25, and FPSB.25 respectively. The fermentation process ameliorated the aroma of fruit juice on account of several secondary metabolites with aromatic properties that were produced [50]. The results indicated that FPSB.25, a mixture of LAB strains *L. plantarum* and *L. salivarius* with *S. Boulardii*, had the potential to improve the overall acceptability to the same extent as FPB.25.

Here, probiotic fermented fruit juices (PFJ) were developed using lactic acid bacteria strains and *S. boulardii* CNCM I-745 as starter culture. The results showed that mixed fruit juice (MFJ) was a suitable substrate to promote the rapid growth and high survival rate of lactic acid bacteria and *S. boulardii*. All three of the probiotic fermented juices (FPB.25, FSB.25, and FPSB.25) exhibited antioxidant and antibacterial activities against *Salmonella* Typhi DMST 22842, which could be the result of volatile compounds from the fermentation-based biotransformation. The sensory attributes based on a 9-point hedonic scale showed that FPB.25 received the highest score (higher 7), demonstrating that the panelists preferred this beverage. Taken together, PFJ could meet present consumer demands regarding natural, fruit-based fermented beverages. Further studies will be needed to evaluate the effects of future beverage products on consumer health.

## Figures and Tables

**Table 1:** Physicochemical properties of fermented fruit juice.

Sample	Physicochemical properties				

	Titratable acidity (%)	pH	Alcohol content (%)	Total sugar (g/l)	Reducing sugar (g/l)
MFJ	0.27 ± 0.07^a^	5.55 ± 0.07^a^	-	1825.81 ± 12.33^a^	0.80 ± 0.03^a^
FP	0.96 ± 0.04^b^	3.74 ± 0.04^b^	-	1255.81 ± 14.43^b^	0.34 ± 0.02^b^
FS	1.08 ± 0.07^b^	3.77 ± 0.06^b^	-	804.98 ± 17.50^d^	0.24 ± 0.01^de^
FPB.25	0.93 ± 0.04^b^	3.76 ± 0.05^b^	0.29 ± 0.05^a^	654.15 ± 3.82^e^	0.29 ± 0.01^bc^
FSB.25	1.11 ± 0.04^b^	3.77 ± 0.06^b^	0.36 ± 0.02^a^	950.81 ± 1.44^c^	0.27 ± 0.02^cd^
FPSB.25	1.07 ± 0.06^b^	3.78 ± 0.07^b^	0.31 ± 0.02^a^	1256.65 ± 12.33^b^	0.22 ± 0.01^e^

MFJ, Mixed fruit juice control; FP, fermented with 1% *L. plantarum* (P); FS, fermented with 1% *L. salivarius* (S); FPB.25, fermented with 1% *L. plantarum* (P) and 0.25% *S. boulardii* (B); FSB.25, fermented with 1% *L. salivarius* (S) and 0.25% *S. boulardii* (B); FPSB.25, mixed FPB.25 and FSB.25 at a ratio of 1:1. Results expressed as means ± SD (*n* = 30). Values with different superscripts in the same column represent statistical data significance (*p* < 0.05).

-, Not detectable.

**Table 2:** MIC and MBC values.

Concentration (mg/ml)	Salmonella Typhi DMST 22842						

	Gentamicin	MFJ	Probiotic fermented fruit juice (PFJ)				

			FP	FS	FPB.25	FSB.25	FPSB.25
MIC	0.039	-	500	500	250	250	250
MBC	0.078	-	500	500	500	500	500

MFJ, Mixed fruit juice control; FP, fermented with 1% *L. plantarum* (P); FS, fermented with 1% *L. salivarius* (S); FPB.25, fermented with 1% *L. plantarum* (P) and 0.25% *S. boulardii* (B); FSB.25, fermented with 1% *L. salivarius* (S) and 0.25% *S. boulardii* (B); FPSB.25, mixed FPB.25 and FSB.25 at a ratio of 1:1.

- No MIC and MBC values were observed due to the lack of antibacterial effect.

**Table 3:** Volatile compounds identified by GC-MS analysis.

Chemical class	Volatile compounds	RT (min)	Odor description	Percentage relative peak area			

				MFJ	Probiotic fermented fruit juice (PFJ)		

					FPB.25	FSB.25	FPBS.25
Alcohols	3-methyl-1-butanol	9.4810	Sweet, alcohol	-	40.3	16.2	41.9
	1-hexanol	13.2363	-	2.5	10.8	21.1	16.8
	2-phenylethanol	25.9774	Roses, honey	-	4.1	1.1	4.2
Aldehyde	Acetaldehyde	2.5754	Pungent	-	2.4	2.2	3.5
Acid	Acetic acid	15.7251	Sour, acid	-	9.2	-	6.4
Esters	Ethyl acetate	3.4763	Pineapple, fruity	72.0	19.3	29.2	26.6
	Ethyl 2-methylbutyrate	5.7967	Fruity	3.6	1.2	1.0	1.4
	Ethyl hexanoate	10.1617	Sweet, fruity	-	2.0	2.1	2.3
	Ethyl lactate	12.9595	Fruity	-	2.5	1.5	2.3
	Ethyl decanoate	20.3464	-	-	0.6	0.3	1.0
Ketone	3-hydroxy-2-butanone	11.4782	Butter	-	12.9	22.9	22.5
Phenol	2,4-di-tert-butylphenol	33.3645	-	81.0	35.1	45.5	53.2
Terpenes	Linalool	18.0575	-	13.5	7.2	9.4	10.1
	β-Damascenone	24.2188	Sweet, fruity	-	1.5	2.8	2.3

MFJ, Mixed fruit juice control; FPB.25, fermented with 1% *L. plantarum* (P) and 0.25% *S. boulardii* (B); FSB.25, fermented with 1% *L. salivarius* (S) and 0.25% *S. boulardii* (B); FPSB.25, mixed FPB.25 and FSB.25 at a ratio of 1:1. Odor descriptions were cited from www.flavornet.org and recent reports.

Percentage relative peak area of volatile compounds is expressed as (compound peak area/total compounds peak area) ×100.

-, Not detectable.
